# Penile metastasis from primay mucinous adenocarcinoma of bladder

**DOI:** 10.4103/0970-1591.33732

**Published:** 2007

**Authors:** Siddalingeshwar Neeli, Vikram Prabha, Sharan Alur, Prakash Malur

**Affiliations:** Department of Urology, KLES Hospital and J.N. Medical College, Belgaum, India; * Department of Pathology, KLES Hospital and J.N. Medical College, Belgaum, India

**Keywords:** Adenocarcinoma, penile metastasis, urinary bladder neoplasm

## Abstract

Primary adenocarcinoma of the urinary bladder is not common. Though penile metastases from transitional cell carcinoma are reported, such metastases from adenocarcinoma of urinary bladder is unknown. We report a 55-year-old male having penile metastasis from primary mucinous adenocarcinoma of bladder.

## INTRODUCTION

Primary adenocarcinoma constitutes 0.5-2% of all urinary bladder malignancies and mucinous adenocarcinoma constitutes around 15% of them.[1,2] Penile metastases from primary bladder carcinoma are rare. We report a case of penile metastasis from primary mucinous adenocarcinoma of urinary bladder.

## CASE REPORT

**Figure 1:**
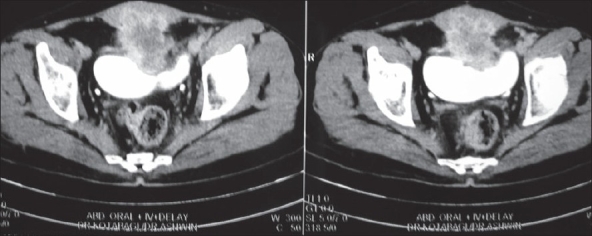
CT scan demonstrating anterior bladder wall growth infi ltrating recti muscles

**Figure 2:**
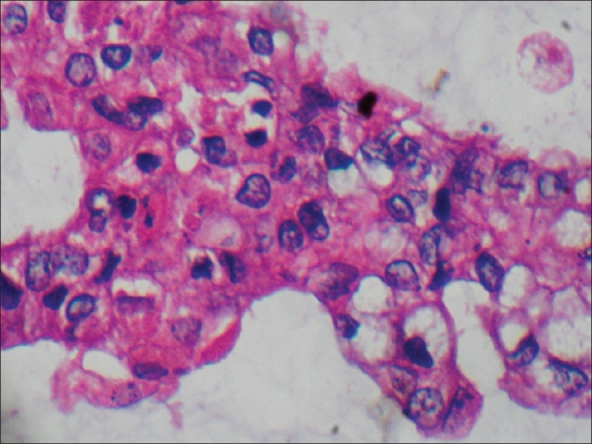
Histopathology of bladder tumor showing malignant cells with atypical nuclei and few signet-ring cells. Background has mucoid material. (H&E, ×40)

A 55-year-old male, chronic smoker, presented with recurrent episodes of painless hematuria for two months. He had undergone cystolithotomy five years back. Physical examination revealed an infraumbilical scar with induration. On cystoscopy, a broad-based solid growth was seen at the dome of the bladder extending onto the anterior bladder wall. Rest of the bladder mucosa and ureteral orifices appeared normal. Biopsy of the growth suggested mucinous adenocarcinoma of the urinary bladder. Computed tomography revealed a growth involving the dome and anterior wall of the bladder extending extravesically and involving the rectus muscles [Figure 1]. Chest X-ray and bone scintigraphy showed no metastasis. Patient underwent radical cystoprostatectomy with en masse excision of the earlier scar and the lower recti muscles with urinary diversion in the form of ileal conduit. Histopathology revealed mucinous adenocarcinoma of the urinary bladder [Figure 2] infiltrating the recti muscles. Cut margins were negative and prostate showed benign hyperplastic changes. The dissected lymph nodes were free of metastasis.

**Figure 3:**
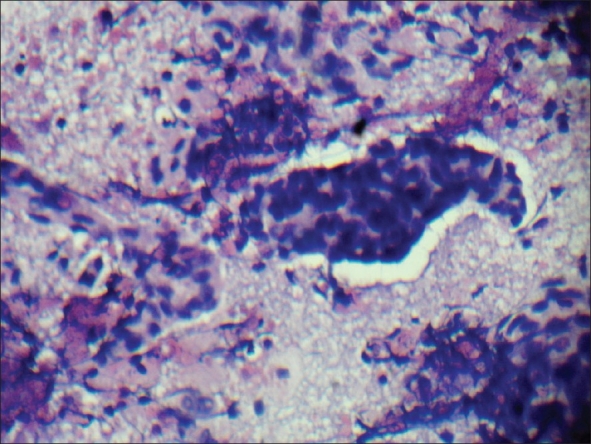
FNAC of penile nodule showing clumps of atypical cells in mucoid background. (H&E, ×20)

**Figure 4:**
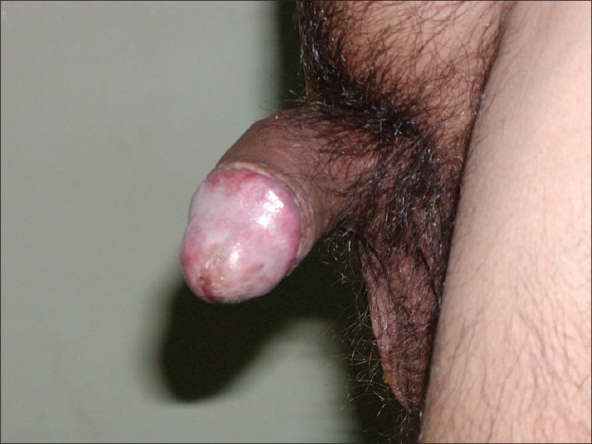
Priapism secondary to malignant infiltration

Six months later the patient presented with pain in the penile shaft. On examination, a nodule was felt in the corporal body. Fine needle aspiration biopsy of the nodule revealed metastasis from mucinous adenocarcinoma [Figure 3]. Repeat metastatic workup was negative for other systemic spread of disease. Patient was advised to undergo total penectomy, but he refused further treatment. One month later he presented with severe pain in the penis not responding to NSAIDs. On examination he had priapism secondary to penile metastases [Figure 4]. Pain was managed with narcotic analgesics. He died of lung and brain metastases after four months.

## DISCUSSION

Primary adenocarcinomas of the bladder are not common tumors. They are commonly reported in cases of extrophy bladder, enterocystoplasty and in endemic areas of bilharziasis.[3–5] Several theories exist regarding the pathogenesis of these tumors, the most common being their origin from metaplastic changes in the potentially unstable epithelium.

Grignon *et al.* classified vesical adenocarcinoma into (1) enteric (papillary), (2) mucinous, (3) signet ring, (4) adenocarcinoma not otherwise classified and (5) mixed.[6] Zaghloul *et al.*, in their large series of 192 patients with vesical adenocarcinoma, had mucinous variety in 14.6% of their cases.[2] Radical cystoprostatectomy and pelvic lymphadenectomy with or without postoperative radiotherapy is the treatment modality employed in the two largest published series.[5,7]

Penile metastases occur in very rare cases. The primary site is the urinary bladder in 30-35%, prostate in 30%, rectosigmoid in 13%, kidneys in 8-10% and testes in 5%.[8] Metastases from the mucinous variety of vesical adenocarcinoma has not been reported in the literature.

Malignant neoplastic lesions spread to the corpora cavernosa by direct extension, retrograde venous or lymphatic transport and arterial embolism.[9] Thrombosis or obstruction of the corpora or irritation of the neural pathways by the metastatic tumor results in priapism.[10]

Penile metastases indicate advanced disease with an average survival of 3.9 months from the time of diagnosis.[8] Total penectomy offers the best chances of survival.[8] In patients with priapism who have a short life expectancy, conservative treatment can be a reasonable choice.

The uniqueness of the present case is it being a rare variety of bladder cancer and later presenting as penile metastasis. Since the tumor had extravesical spread and had infiltrated abdominal wall muscles, we feel retrograde venous spread as the cause for penile metastasis. The aggressive tumor behavior resulted in rapid progression of the disease and eventual mortality.
